# Sodium Hydroxide Pretreatment as an Effective Approach to Reduce the Dye/Holes Recombination Reaction in P-Type DSCs

**DOI:** 10.3389/fchem.2019.00099

**Published:** 2019-02-27

**Authors:** Matteo Bonomo, Nadia Barbero, Gaia Naponiello, Marco Giordano, Danilo Dini, Claudia Barolo

**Affiliations:** ^1^Department of Chemistry, University of Rome “La Sapienza”, Rome, Italy; ^2^Department of Chemistry and NIS Interdepartmental Centre and INSTM Reference Centre, University of Turin, Torino, Italy; ^3^ICxT Interdepartmental Centre, Torino, Italy

**Keywords:** squaraines, p-type dye-sensitized solar cells, NIR Absorption, NaOH pretreatment, recombination reaction, electrochemical impedance spectroscopy

## Abstract

We report the synthesis of a novel squaraine dye (VG21-C12) and investigate its behavior as p-type sensitizer for p-type Dye-Sensitized Solar Cells. The results are compared with O4-C12, a well-known sensitizer for p-DSC, and sodium hydroxide pretreatment is described as an effective approach to reduce the dye/holes recombination. Various variable investigation such as dipping time, dye loading, photocurrent, and resulting cell efficiency are also reported. Electrochemical impedance spectroscopy (EIS) was utilized for investigating charge transport properties of the different photoelectrodes and the recombination phenomena that occur at the (un)modified electrode/electrolyte interface.

## Introduction

The development of a green, sustainable, renewable, and environmental-friendly technology for energy production rapidly have gained relevance due to the well-known global warming issue (Reddy et al., 2014). Energy conversion from solar light is the more feasible and widely exploitable of those technologies. Recently, beside silicon-based devices, different technologies [e.g., Dye-Sensitized Solar Cells, DSCs (Hagfeldt et al., 2010; Ning et al., 2010; Thomas et al., 2014), Organic PhotoVoltaics, OPV (Kippelen and Brédas, 2009), Perovskite Solar Cells, PSC (Park, 2015; Green and Ho-Baillie, 2017), Copper Zinc Tin Sulfide-based solar cells, CZTS (Ravindiran and Praveenkumar, 2018)…] have achieved both scientific and commercial visibility. Dye-Sensitized Solar Cells (DSCs) could be effectively employed in both outdoor and indoor applications. Since their first appearance in 1991, (O'Regan and Grätzel, 1991) scientists have focused their efforts on n-type DSC, where a TiO_2_-sensitized electrode behaves as photoanode, reaching an overall efficiency up to 14% (Yella et al., 2011; Mathew et al., 2014; Kakiage et al., 2015).

This value could be considered as the upper threshold for single junction devices, nevertheless, coupling a photoanode to a photocathode in a sandwiched device (t-DSCs) (Xu et al., 2015) has been reported to better the photoconversion performances. The photocathodes (p-type DSC if considered as single junction device) research is still in an early stage (Bonomo and Dini, 2016) and the higher efficiency, so far reported, for a p-DSC is one order of magnitude lower than the corresponding n-type counterpart (Perera et al., 2015). The obtainment of a more efficient p-DSCs is strictly related to successfully (i) improve the electronic properties of NiO photocathodes to faster the charge transport throughout the electrode (Hsu et al., 2012); (ii) minimize the recombination phenomena (both D^*^/h^+^ and I^−^/h^+^, with the holes formally localized on the Ni^3+^ defective, surface sites) resulting in a higher photons conversion (D'Amario et al., 2015); (iii) develop materials (Bai et al., 2013; Jiang et al., 2016), i.e., sensitizers and/or electrolytes (Perera et al., 2015; Bonomo et al., 2018a) specifically and thoughtfully designed for p-type DSCs; (iv) efficiently implement materials developed for n- and p-DSC as well.

The improve in NiO photocathodes properties (i) could be addressed by using an innovative deposition (Awais et al., 2015) or a sintering procedure (Novelli et al., 2015) to obtain more porous and conductive electrodes. The recombination phenomena (ii) could be partially solved by moving the acceptor group far from the electrode and, at the same time, in a way to effectively cover the photocathode surface to minimize the undesired interaction with iodide (Weidelener et al., 2012; Wood et al., 2014; Perera et al., 2015); various sensitizers (iii) [i.e., triphenyl-ammine (Pham et al., 2017) squaraines (Saccone et al., 2016) iso-indigo (Thoi et al., 2013; Ameline et al., 2015) pyranic (Bonomo et al., 2017a) quinonic (Bonomo et al., 2017d), or diketopyrrolic dyes (Farré et al., 2017)…] have been effectively employed in p-DSC in the last years, while less efforts were devoted to the development of alternative electrolytes (Odobel et al., 2010; Nikolaou et al., 2017).

Different approaches have been recently reported to minimize the recombination reactions. Chenodeoxycholic Acid (CDCA) has been added, as widely made for n-DSC, to the sensitization solution to efficiently cover the unbounded Ni^3+^ sites but it could also compete with dye during the sensitization leading to a lower dye-loading. Odobel et al. (Favereau et al., 2017) have tackled this drawback by adding the CDCA to the electrolyte solution. This approach did not lessen the dye-loading but the DSCs long-term stability was still affected by the CDCA presence. D'Amario et al. (2015, 2017) have exploited a different approach based on a thermal or chemical (with NaBH_4_) pretreatment to reduce the amount of free Ni^3+^ surface sites. Both pretreatments have led to higher open circuit voltage and FF values due to the minimization of the recombination phenomena (directly correlated to the reduce number of Ni^3+^ defects). Unexpectedly, the overall conversion efficiency was not affected by the concurrent decrease of the dye-loading.

Some of us have previously reported the effect of a soda pretreatment on a NiO electrode surface on the photoelectrochemical properties of squaraine-based p-type DSC (Bonomo et al., 2018b). The chemisorption of the hydroxyl moieties on the NiO surface has been shown to lower the dye-loading leading to a lower photocurrent density. On the other hand the Electrochemical Impedance Spectroscopy (EIS) measurements have suggested that NaOH-modified electrodes are less prone to recombination phenomena (i.e., higher recombination resistance has been produced in comparison to pristine photocathodes). Moreover, the soda pretreatment has resulted in a higher long-term stability of the devices while less pronounced decreases in the J_SC_ value were associated to longer side alkyl chain on the squaraines.

**Figure 1:**
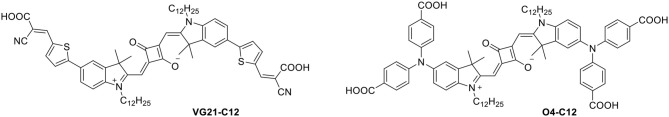
Structures of VG21-C12 and O4-C12.

Here we introduce the synthesis and the applications of a new squaraine dye (VG21-C12) as a sensitizer for p-DSC in comparison to the derivative O4-C12 as a reference (Figure 1) (Bonomo et al., 2018b).

It is worth to mention that, among the previously reported O4-CX series, the latter is not the most performing dye, but it has been chosen as reference to overlook the effect of the same alkyl chain (C12).

## Experimental

### Materials and Methods

All the chemicals were purchased from Sigma Aldrich and were used without any further purification.

All microwave reactions were performed in single-mode Biotage Initiator 2.5. TLC were performed on silica gel 60 F254 plates.

LC-HRMS analyses were accomplished with an Ultimate 3000 HPLC instrument (Dionex, Milan, Italy) coupled to an LTQ Orbitrap instrument at a resolution of 30,000 (500 m/z FWHM) in FTMS mode (Thermo Scientific, Rodano, Italy) with an APCI interface.

UV–Vis spectra were recorded on a Shimadzu UV-1700 Pharma Spec using different solvents in order to investigate the solvatochromic behavior of the symmetrical squaraine VG21. A stock solution in absolute ethanol (EtOH) was prepared and diluted solutions in ethanol (EtOH), methanol (MeOH), acetonitrile (ACN), tetrahydrofuran (THF), dichloromethane (DCM), and dimethyl sulfoxide (DMSO) were analyzed.

Fluorescence measurements in steady state mode were acquired using a Horiba Jobin Yvon Fluorolog 3 TCSPC fluorimeter equipped with a 450-W Xenon lamp and a Hamamatsu R928 photomultiplier. Diluted solutions ([dye] < 0.01 mM) with absorbance around or lower of 0.1 units were used to avoid the presence of aggregates.

The absolute quantum yield of VG21-C12 in ethanol was determined combining Quanta-ϕ with Fluorolog 3 and De Mello method. The reported value is the average of three measurements using three different VG21 solutions. The excitation wavelength was set to 640 nm, excitation and emission slits were 6 and 12 nm, respectively, and the emission was acquired from 660 nm to 850 nm.

Fluorescence lifetimes were measured by the time correlated single photon counting method (Horiba Jobin Yvon) using a 636 nm Horiba Jobin Yvon NanoLED as excitation source and an impulse repetition frequency of 1 MHz positioned at 90° with respect to a TBX-04 detector. Lifetime was calculated using DAS6 decay analysis software. The goodness of the fit was assessed by the chi-squared value of <1.05 and a residual trace that was symmetric about the zero axes.

^1^H NMR (200 MHz) spectra were recorded on a Bruker Avance 200 NMR.

### Synthesis

The intermediates 5-bromo-2,3,3-trimethyl-3H-indole, 5-bromo-1-dodecyl-2,3,3-trimethyl-3H-indol-1-ium iodide and squaraine **Br-C12** were prepared as described in literature (Barbero et al., 2015).

Synthesis of **3**: Squaraine **Br-C12** (200 mg, 0.22 mmol), 5-formyl-2-thienylboronic acid (206 mg, 1.32 mmol), [PdCl_2_(dppf)]CH_2_Cl_2_ (36 mg, 0.04 mmol), potassium carbonate (304 mg, 2.2 mmol), and a mixture of toluene and methanol (1:1, 4 mL) were introduced in a dry reaction vial which was sealed with a crimp cap and heated in a microwave system at 70°C for 20 min. The reaction mixture was then extracted with water and dichloromethane, the organic phase was treated with Na_2_SO_4_, filtered off and the solvent was removed under vacuum. The product was further treated with diethyl ether and the resulting precipitate afforded product **3** as a dark/gray powder (200 mg, yield = 94%).

HRMS (ESI +.ve) calcd for [M]^+^ 953.5319, found [M]^+^ 953.5369 and [M+Na] ^+^ 975.5189.

^1^H-NMR: (CDCl_3_) δ ppm 9.89 (s, 2H), 7.75 (d, *J* = 4.0 Hz, 2H), 7.665 (d, *J* = 2.0 Hz, 4H), 7.41 (d, *J* = 4.0 Hz, 2H), 7.02 (d, *J* = 4.0 Hz, 2H), 6.03 (s, 2H), 4.02 (m, 4H), 1.84 (s, 12H), 1.33 (m, 40H), 0.87 (t, *J* = 12.0 Hz, 6H).

Synthesis of **VG21-C12**: Compound **3** (100 mg, 0.1 mmol) and cyanoacetic acid (34 mg, 0.4 mmol) were introduced in a dry reaction vial which was sealed with a crimp cap with dry acetonitrile (5 ml). Piperidine (0.4 mmol, 40 μl) was then added and the reaction mixture was heated in a microwave system at 100°C for 60 min. The reaction mixture was then washed with methanol which afforded the precipitation of **VG21-C12** as a dark powder (90 mg, yield = 83%).

HRMS (ESI +.ve) calcd for [M]^+^ 1087.5436, found [M]^+^ 1087.7643.

^1^H-NMR: (CDCl_3_) δ ppm 8.21 (s, 2H), 7.67–7.57 (m, 6H), 7.36 (d, *J* = 4.0 Hz, 2H), 6.98 (d, *J* = 10.0 Hz, 2H), 5.95 (s, 2H), 3.97 (m, 4H), 1.73 (s, 12H), 1.23 (m, 40H), 0.77 (t, *J* = 12.0 Hz, 6H).

The compound solubility in CDCl_3_ was too low to record a ^13^C NMR spectrum.

UV-Vis: λ_max_ (EtOH) 690 nm.

### Cell Assembly and Characterization

NiO photocathodes (thickness = 3 μm) were obtained by screen-printing and oven sintering of a NiO slurry (named P1, containing 1 ml of glacial acetic acid) as reported in previous works (Bonomo et al., 2017c). Photocathodes were sensitized by dipping them in an ethanol solution of VG21-C12 (0.3 mM) for different amount of time, i.e., 2 h and 16 h (Over Night, ON). When applied, Soda (NaOH) pretreatment consisted in dipping the electrode in a sodium hydroxide aqueous solution (0.1 M) for 2 h. The photocathodes were then rinsed with deionized water to eliminate possible adsorbed molecules. The sensitized electrodes were then coupled in a sandwich configuration with a Pt-based counter-electrode (De Rossi et al., 2013) using Surlyn® as both sealant and spacer. A LiI/I_2_-based electrolyte (HSE electrolyte from Dyesol®) was injected by back-vacuum technique through a hole in the Surlyn mask. The hole was then closed by a drop of a UV-curable resin (TB3035B from ThreeBond®).

The cells were let stabilized for 24 h before testing them. Characteristic curves current-potential (JV) were recorded using a solar simulator (class A) at 1,000 W^*^m^−2^ with artificial solar spectrum AM 1.5 G. The curves of IPCE (Incident Photon-to-current Conversion Efficiency) were recorded using a computer-controlled set-up consisting of a Xe lamp (Mod.70612, Newport) coupled to a monochromator (Cornerstone 130 from Newport), and a Keithley 200 2420 light-source meter. The determination of the electrochemical impedance spectra (EIS) was performed with AUTOLAB PGSTAT12® (Metrohm) at the condition of open circuit potential and under illumination at 1,000 W^*^m^−2^ with a sun simulator producing the AM1 1.5G spectrum. The sinusoidal perturbation of the potential had an amplitude of 10^−2^ V and it was applied within the frequency range 10^−1^-10^5^ Hz. In order to calculate the electrochemical cells parameters derived from the analysis of the EIS profiles, the impedance spectra were fitted using the software Z-View 2.1.

## Results and Discussion

**Scheme 1:**
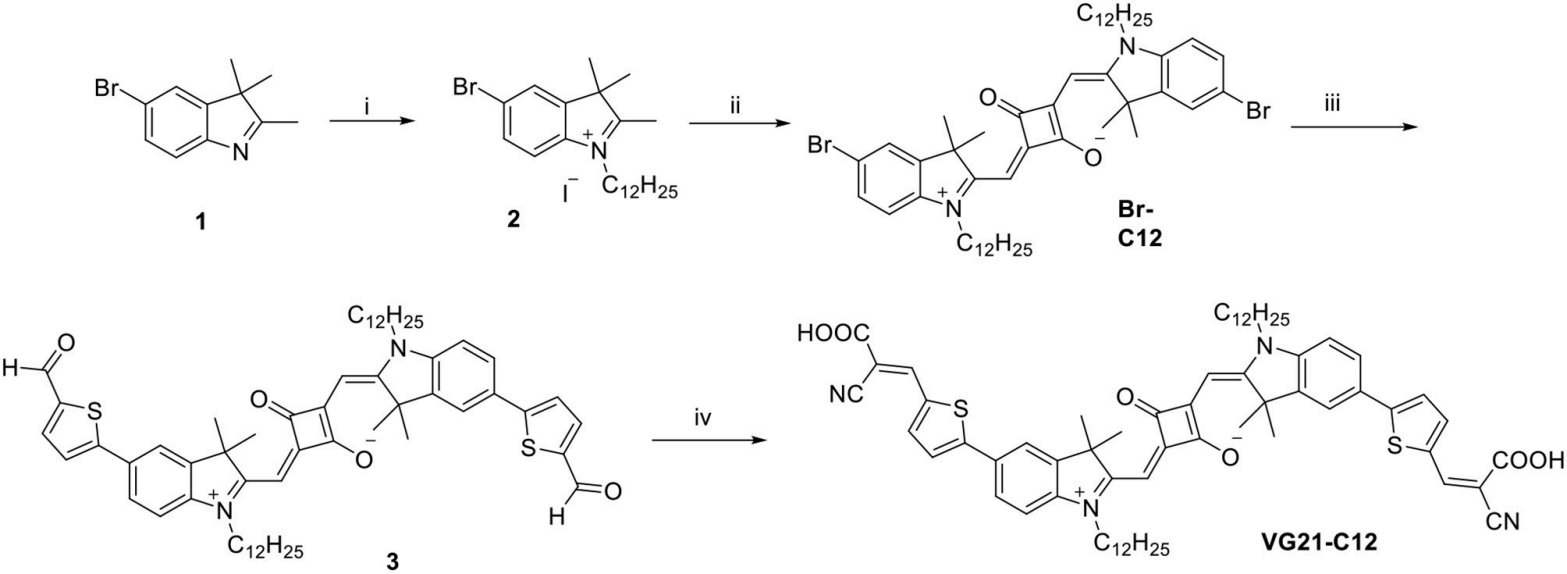
Scheme of the synthesis of VG21-C12. Reagents and conditions: (i) 1-iodododecane, acetonitrile, microwave, 155°C, 60 min; (ii) squaric acid, toluene/n-butanol (1:1), MW, 160°C, 30 min; (iii) 5-formyl-2-thienylboronic acid, [PdCl_2_(dppf)]·CH_2_Cl_2_, K_2_CO_3_, toluene/MeOH (1:1), microwave, 70°C, 20 min; (iv) cyanoacetic acid, piperidine, acetonitrile, microwave, 100°C, 60 min.

The synthesis of the symmetrical squaraine **VG21-C12** is reported in Scheme 1. The squaraine precursor **Br-C12** was obtained starting from 4-bromophenylhydrazine hydrochloride as reported in literature (Barbero et al., 2015). Compound **3** was obtained in high yield by a microwave-assisted Suzuki coupling with 5-formyl-2-thienylboronic acid with slight modifications from a literature procedure (Shi et al., 2011). This compound was further condensed, by microwave reaction, with cyanoacetic acid to afford squaraine **VG21-C12**.

**Figure 2:**
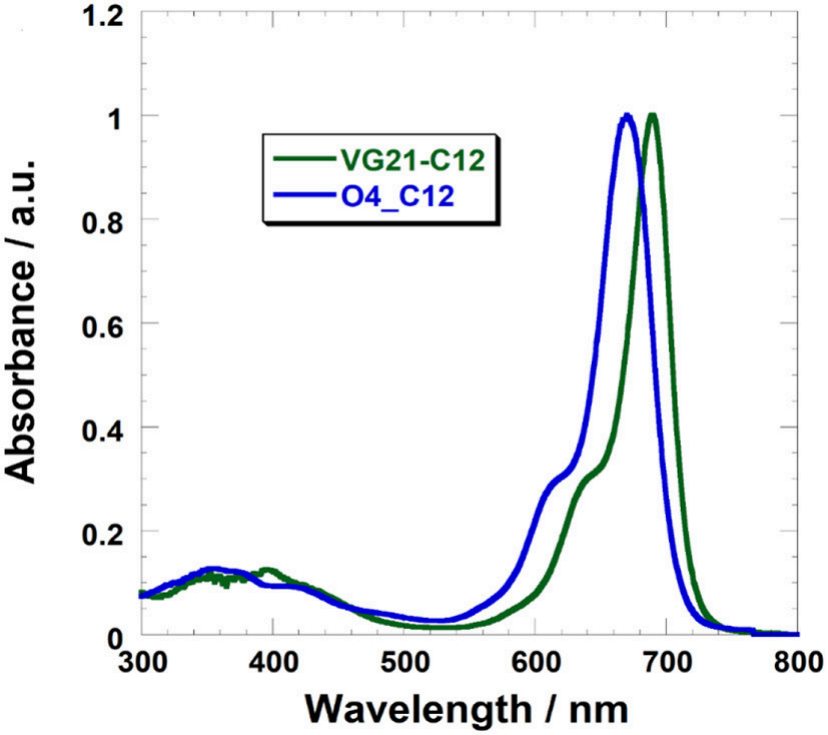
UV-Vis absorption spectra of VG21-C12 (green) and O4-C12 (blue) in ethanol solution.

The absorption of VG21-C12 (λ_max_ = 690 nm in ethanol) is red-shifted compared to O4-C12 (λ_max_ = 670 nm) (Figure 2).

VG21-C12 shows a narrow absorption band in the NIR with the hypsochromic shoulder typical of polymethine dyes. Table 1 shows the main photophysical properties of VG21-C12 in different solvents. VG21-C12 is nearly non-solvatochromic: as general trend, neither the absorption maxima nor the band shapes are affected by the solvent polarity or proticity. Moreover, the small Stokes shift (5–10 nm) values indicate that a moderate geometry change occurs from the ground to the excited state. VG21-C12 is characterized by a short lifetime (1.47 ns in ethanol) and a considerable fluorescence quantum yield (41% in ethanol).

**Table 1:** Selected photophysical properties of VG21-C12 in different solvents.

**Solvent**	**λ_**abs**_ (nm)**	**λ_**em**_ (nm)**	**Stokes shift (nm)**
THF	701	703	2
DCM	696	699	3
DMSO	708	714	6
ACN	695	700	5
EtOH	690	701	11
MeOH	691	701	10

The NIR-type absorption of VG21-C12 is extremely meaningful for the implementation of this sensitizers in tandem [both anode and cathode photoactive (Nattestad et al., 2016)] or in co-sensitized [electrode sensitized with two or more dyes at once (Perera et al., 2005; Clifford et al., 2011)] devices. As a matter of fact, the more broadly investigated sensitizers for DSCs absorb light between 350 and 600 nm whereas they lack a strong absorption at longer wavelengths. Therefore, the co-sensitization of the latter with a dye absorbing in the Near Infra-Red (NIR) region (as VG21-C12 is) could be a profitable approach to exploit the whole solar irradiation spectra. Similarly, the complementarity of the absorption spectra of two different sensitizers is a key factor to obtain highly efficient tandem DSCs.

From a theoretical point of view VG21-C12 should not be suitable as sensitizer in p-type DSCs because the presence of a cyanoacrylate group localizes the charge density close to the electrode surface leading to an (unwanted) promotion of the D^*^/h^+^ recombination reaction, being D^*^ the excited form of the dye and h^+^ the hole injected in the valence band of the photocathode (Favereau et al., 2017). In the present work we demonstrate that this evidence is not completely relevant when the electrode is pretreated with soda.

**Figure 3:**
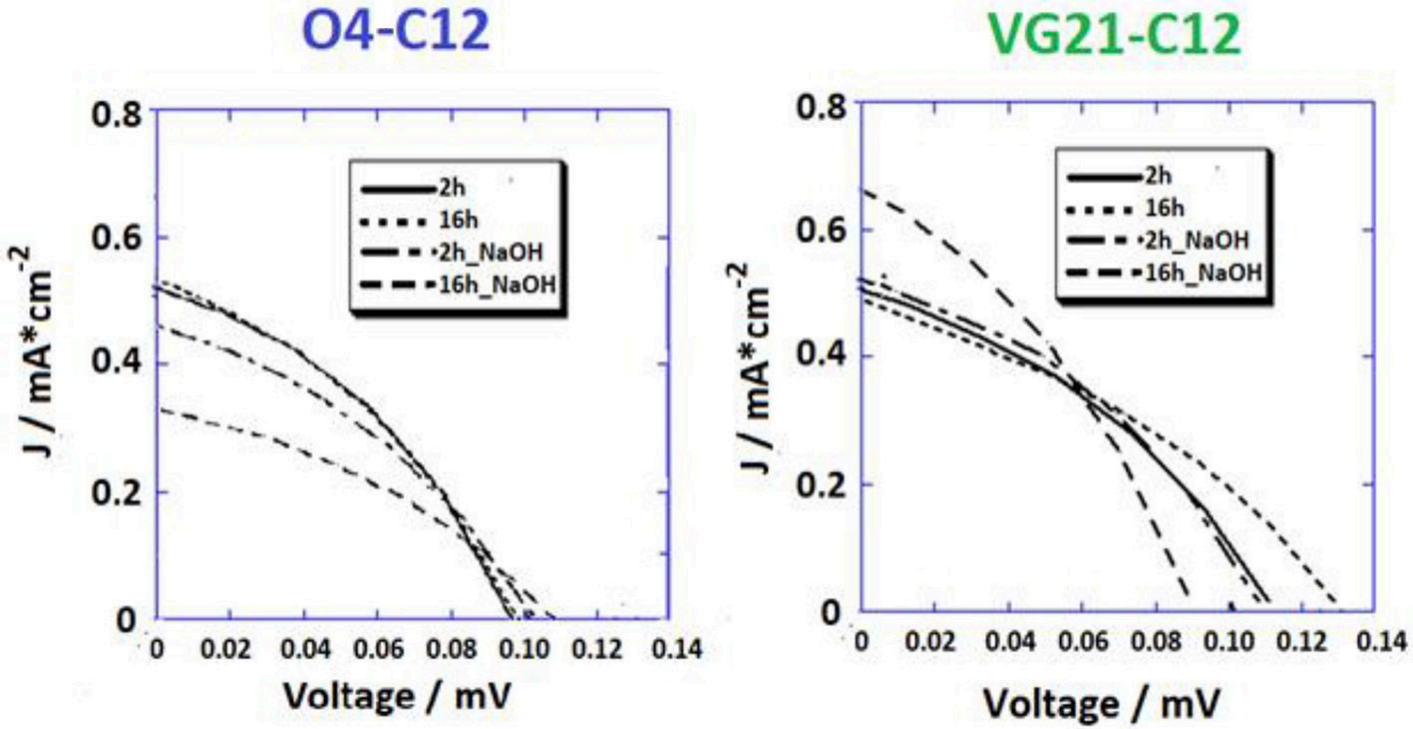
JV profiles of NiO-based DSCs sensitized with two different squaraine dyes, i.e., O4-C12 (on the **left**) and VG21-C12 (on the **right**).

The JV curves of pristine NiO photocathode sensitized with both the VG21-C12 and O4-C12 have been analyzed (Figure 3). Two different sensitization times (2 and 16 h) were investigated with the longer corresponding to an over-night (ON) sensitization. Shorter dipping time are optimal for squaraines without triphenyl-ammine group because sensitization time longer than 2 h could lead to the formation of intermolecular adducts [*H* or *J* forms (Lee et al., 2016)] that heavily limit the charge injection efficiency (Bonomo et al., 2016). As a matter of fact, this drawback was not present when squaraine bearing one or two triphenyl-ammine moieties were employed as sensitizers [e.g., O4-CX (Langmar et al., 2017), DS_XX series (Bonomo et al., 2017e)] for which a higher dye-loading value were achieved by longer dipping time plateauing after 16 h.

Higher dye-loading and wider NiO surface coverage was achieved with VG21-C12 due to its smaller size in comparison to the O4-C12, showing a higher open circuit potential value. Nevertheless, we have recently shown that higher V_OC_ could arise from the presence of available (i.e., unbounded) Ni^3+^ sites at the electrode/electrolyte interface (Bonomo et al., 2018a). Remarkably, the Fill Factor (FF), which is related to recombination phenomena, did not evidence any increase in the devices herein described (Nakasa et al., 2005). A wider electrode coverage, along with a sensible increase in the open circuit potential value, was achieved by extending the sensitization time up to 16 h (ON). On the other hand, the current density was affected by a slight decrease in presence of the VG21-C12, most likely because of aggregation phenomena (*H* or *J* adducts) tacking place when using VG21-C12 for long sensitization time. The sensitizer aggregation was not observed when the VG21-C12 was replaced by O4-C12 due the bulkier structure of this dye. Similar trend in the experimental FF values where obtained. In presence of VG21-C12, the FF was affected by the formation of bimolecular adducts, promoting charge transfer processes and hampering the charge injection from the dye to the NiO valence band.

Overall, the higher V_OC_ was negatively counterbalanced by lower FF and J_SC_ leading to a 10% decrease in the VG21-C12 device efficiency. Conversely, the O4-sensitized device was characterized by a simultaneous increase of the J_SC_ and FF producing a remarkable 16% increase in the conversion efficiency (Table 2).

**Table 2:** Photoelectrochemical parameters of VG21-C12 and O4-C12-sensitized NiO-based DSCs.

**Dye**	**Sens. time (h)**	**V_OC_ (mV)**	J_SC_ (mA*cm^−2^)	**FF (%)**	**η (%)**
VG21-C12	2	116	0.459	36.7	0.020
O4-C12	2	100	0.494	37.2	0.019
VG21-C12	16	130	0.410	35.2	0.018
O4-C12	16	105	0.518	39.1	0.022

*Sensitization process was performed for 2 and 16 h*.

The reduction of the unbounded free Ni^3+^ sites concentration at the electrode/electrolyte interface was successfully achieved by a pretreatment of the NiO electrode with sodium hydroxide (NaOH) (Figure 2, Table 3). This is a key feature to consider because the recombination between Ni^3+^ sites and I^−^ anions is a process that heavily affects the photoconversion efficiency in p-type DSCs. Unfortunately, the hydroxyl ions compete with the sensitizer in bounding NiO surface, limiting the dye-loading.

**Table 3:** Photoelectrochemical parameters of VG21-C12 and O4-C12-sensitized NiO-based DSCs.

**Dye**	**Pretreatment time with NaOH (h)**	**Sens. time (h)**	**V_OC_ (mV)**	J_SC_ (mA*cm^−2^)	**FF (%)**	**η (%)**
VG21-C12	2	2	109	0.532	39.1	0.022
O4-C12	2	2	104	0.438	36.9	0.016
VG21-C12	2	16	91	0.616	37.1	0.020
O4-C12	2	16	110	0.311	36.8	0.013

*The photocathode underwent a pretreatment with NaOH. Sensitization process was performed for 2 and 16 h*.

The usage of bulky squaraines has proven that the lower dye-loading is not counterbalanced by a reduced amount of recombination phenomena. The situation is slightly different when VG21-C12 is employed. The presence of Ni^3+^-OH adducts at the NiO surface reduces the dye-loading but, at the same time, strongly prevents D^*^/h+ unwanted charge transfer (i.e., Ni^3+^ sites became less easily available) and hampers the formation of bimolecular adducts because the number of proximal anchoring sites is lower in comparison to the pristine NiO electrode. The undesired charge transfer pathways reduction was reflected in the higher FF value (close to 40%), whereas the latter effect was evidenced by the higher value of J_SC_ with the increase of sensitization time.

The soda pretreatment effect was related to the sensitizer nature. The VG21-C12 electronic properties showing the HOMO level mainly localized on the cyano-acrylic moiety, close to the electrode surface, should prevent its employment as p-type sensitizer. This was clearly evidenced by better experimental results obtained with the O4-C12 untreated device in comparison to the VG21-C12 one. On the other hand, upon treatment with NaOH, the VG21-C12 device has shown higher performance (+38%) in comparison to the O4-C12 one. A high overall photoelectrochemical conversion efficiency for the VG21-C12-sensitized (ON) device was prevented by an anomalous V_OC_ (i.e., 91 mV) value.

IPCE spectra were recorded to confirm the photoelectrochemical parameters measured by JV. IPCE provides the percentage of photons, per wavelength, that are effectively converted into current. A detailed investigation of IPCE profiles could provide meaningful information on the photoelectrochemical behavior of both the semiconductor and the sensitizer, in presence or absence of NaOH. Two distinct peaks (or absorption areas) were depicted in each IPCE spectrum independently on the employed sensitizer. A first peak was centered around 380 nm showing the photocathode photoactivity and a second, broader peak, at longer wavelength correlated to the sensitizer absorption. It is worth to mention that NiO photoactivity is mainly due to the presence of defective Ni^3+^ sites in the photoelectrode matrix and, any shift in the IPCE value of the first peak is, intrinsically, linked to a change in the number of defective sites.

**Figure 4:**
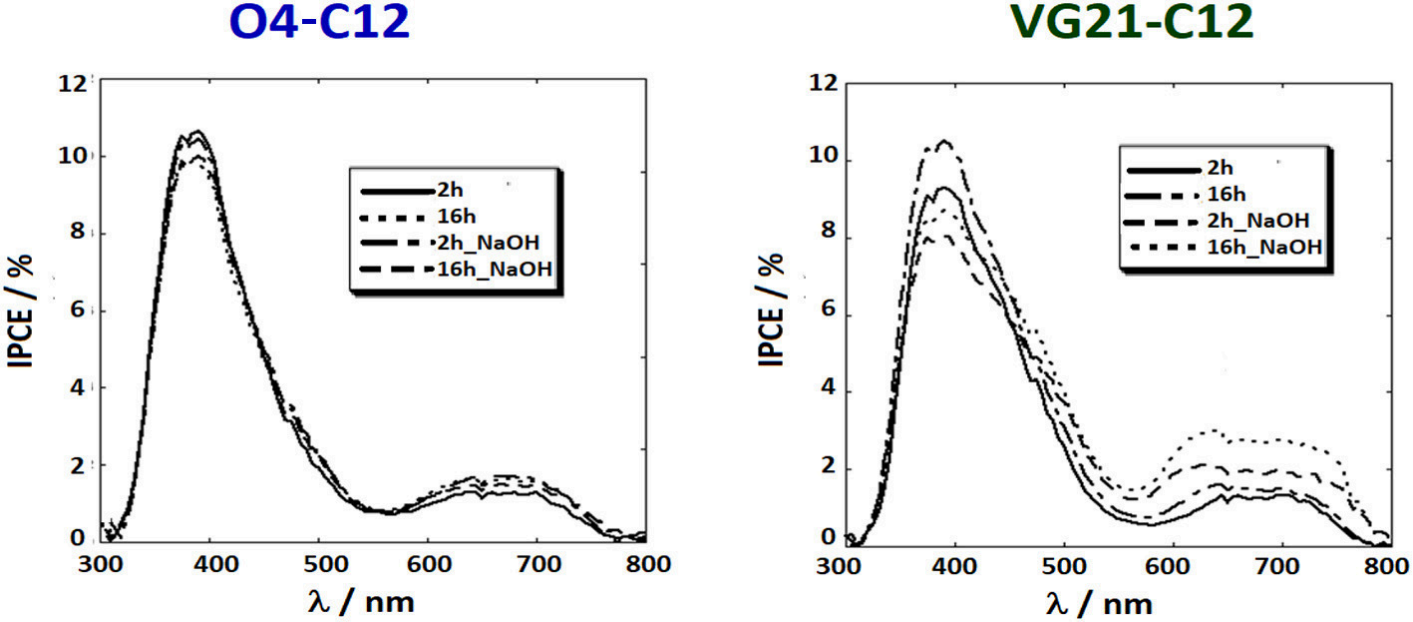
IPCE profiles of NiO-based DSCs sensitized with two different squaraine dyes, i.e., O4-C12 (on the **left**) and VG21-C12 (on the **right**).

IPCE spectra of O4-C12-based devices (Figure 4) were not affected by the sensitization time, while the NaOH-treated devices were depicted by a lower photoactivity of the NiO due to the partial passivation of Ni^3+^ sites. Conversely, the sensitizer peak was slightly increased because of the recombination reaction reduction despite the lower dye-loading. The effect of soda pretreatment was clearer in the VG21-C12 case and depicted by a slight decrement of the first peak along with an increase of the sensitizer peak. A higher value of dye-loading is not feasible due to the competition between OH^−^ and dye in binding the NiO surface. The increase of IPCE profile in the NIR region could most likely be related to the lowering of the D^*^/h^+^ recombination reactions (i.e., fewer injected photons recombine before diffusing throughout the NiO electrode). This effect is more evident for longer sensitization time, i.e., 16 h, while the bimolecular adduct formation was prevented by the presence of the hydroxy moieties on the NiO surface.

**Figure 5:**
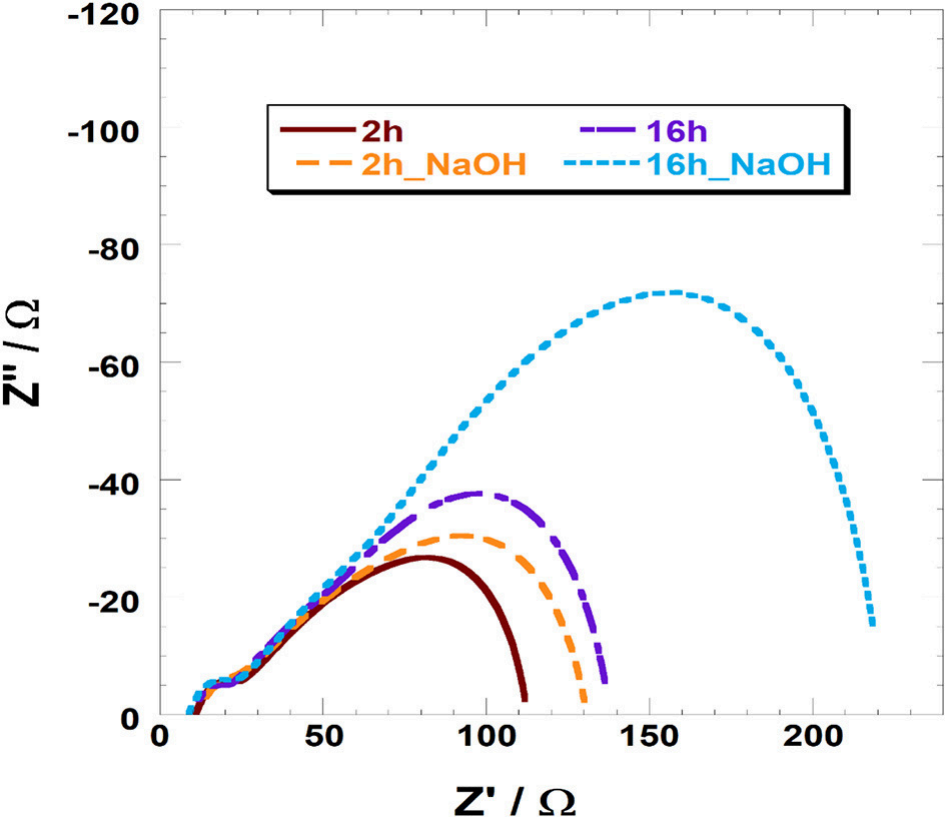
EIS analysis of VG21-C12 based devices.

JV and IPCE profiles show that the soda pretreatment has a beneficial effect onto the photoelectrochemical performances of NiO-based DSCs when a n-type, and relatively small sensitizer, is employed (Table 3). At this regard, electrochemical impedance spectroscopy (EIS) was considered to investigate the charge transport and recombination processes. In Figure 5, we reported the spectra of VG21-C12-based device for different dipping time and with or without the NaOH-pretreatment. In order to obtain useful information on the charge transport and recombination phenomena, the experimental data were interpolated with a proper equivalent circuit specifically designed for analyses of p-type DSCs (Bonomo et al., 2016). The latter is a modified version of the equivalent circuit proposed by Bisquert for n-DSCs (Bisquert, 2002). In EIS spectra, two different semicircles could be highlighted: the high-frequency one is modeled with a resistor/capacitor element and simulate the electrochemical processes occurring at the counter-electrode/electrolyte interface; the second, shaped by the *transmission line* model, simulate both the transport processes through the photocathode and the recombination reactions at the photocathode/electrolyte interface.

The parameters obtained by the fitting procedure of the impedance spectra are reported in Table 4. All devices presented similar values of the charge transfer resistance at the counter-electrode (R_CE_) whereas they differed quite considerably in the terms of transport and recombination resistance (R_t_ and R_rec_, respectively).

**Table 4:** Parameters of VG21-C12-sensitized (un)treated NiO-based DSCs obtained from the fitting of the impedance spectra reported in Figure 5.

**Pretreatment time with NaOH (h)**	**Sens. time (h)**	**R_CE_ (Ω)**	**R_t_ (Ω)**	**R_rec_ (Ω)**	**C_μ_ (mF)**
0	2	7.2 ± 0.3	72 ± 6	104 ± 2	0.43
2	2	6.8 ± 0.5	80 ± 5	134 ± 3	0.41
0	16	6.9 ± 0.3	54 ± 2	176 ± 6	0.38
2	16	6.6 ± 0.4	65 ± 3	322 ± 11	0.36

*The relative error on C_μ_ is lower than 0.01 mF*.

The recorded value of R_t_ is lower for the NiO samples treated with a longer dipping time following a higher dye loading. As a consequence of that, hole injection in the valence band of NiO resulted more effective. The pretreatment with sodium hydroxide leads to an increase in the transport resistance. This evidence is consistent with the low amount of VG21-C12 anchored onto the photocathode because of the competition occurring with the hydroxyl moieties. The differences are relatively low because both soda pretreatment and sensitization involve the surface of the electrode, whereas charge transport taking place throughout the electrode involves mainly the bulk properties of the cathode. The effect of the sensitization and soda pretreatment is more evident in the recombination resistance values: the longer the dipping time the higher the R_rec_ with the latter that increases also when the electrode is modified. As a matter of fact, both the dye and hydroxyl moieties act as passivating agent toward the Ni^3+^ sites (either photogenerated or pre-existing in the as prepared cathode) (Bonomo et al., 2017b).

Straightforwardly, the latter species become less accessible to iodine anions and the recombination reaction between I^−^ and h^+^ (a hole in the VB of NiO) is thus partially hindered. The minimization of the recombination reactions results to be a cooperative effect of both longer dipping time and soda pretreatment (R_rec_ = 322 Ω, see Table 4): on one hand the dipping in NaOH followed by a short sensitization (i.e., 2 h) is not enough to completely passivate Ni^3+^ sites; on the other hand, the sole dipping in dye solution does not assure the whole and homogeneous covering of the surface due to the relative large size of VG21-C12. The chemical capacitance of the electrode, C_μ_, i.e., a parameter which accounts for the rising of a double layer capacitor at the electrode/electrolyte interface confirms this analysis: a larger C_μ_ is consequence of a larger charging of the photocathode surface due to a larger surface concentration of defective Ni^3+^ sites. Both dye and OH^−^ strongly bind these sites and, consequently, diminish the exposed charge localized at the electrode/electrolyte interface.

Fitting of the EIS profiles confirmed the trend of the data obtained from the analyses of the JV curves and IPCE spectra. On the other hand, it is recognized that the soda pretreatment minimizes the reaction of recombination but limits also the uploading of the dye onto the photocathode.

The evidences herein reported show, for the first time, that the so-called n-type sensitizers (i.e., dyes with electronic features matching the requirement for n-DSCs) could be effectively employed in p-type DSC along with a specific pretreatment of the photocathode surface. This opens the way to the implementation of a large library of dyes as efficient sensitizers in p-DSCs.

## Conclusions

In the present work, we have reported the synthesis of a new squaraine dye, VG21-C12, that presents a symmetric structure with a cyano-acrylic unit as an anchoring moiety. Compared to the classical squaraines [i.e., VG1, (Park et al., 2012) VG10 (Park et al., 2014) and VG2 (Galliano et al., 2016) series], the new sensitizer comprises a thiophene unit as an additional spacer. VG21-C12 was employed as sensitizers in p-type DSCs. Different sensitization times were investigated, showing that times longer than 2 h are detrimental for the photoconversion performance of the device, due to the raising of bimolecular (i.e., dye/dye) adducts and leading to lower current density compared to the reference (i.e., O4_C12). Indeed, the higher dye-loading obtained with the VG21 (due to its smaller size) was negatively counterbalanced by the occurrence of D^*^/h^+^ recombination reactions. The latter are substantially minimized when the NiO photocathode was pretreated with an aqueous solution of sodium hydroxide. NaOH partially passivates Ni^3+^ defective sites (i.e., where the holes, h^+^, are localized), leading to higher current density and FF values. Furthermore, NaOH seems to avoid the adducts formation. Photoelectrochemical results were confirmed by EIS analysis: impedance spectra evidenced that longer sensitization time and soda pretreatment minimize recombination phenomena. The present work proves, probably for the first time, that dye-sensitizers initially designed for n-type DSCs can be effectively employed in p-DSCs as well.

## Author Contributions

DD and CB conceived and designed the molecules and the synthesis. NB and MG performed the synthesis and the characterizations. MB and GN prepared the photovoltaic devices and performed the photoelectrochemical analysis. MB and NB work on the writing of the draft. All the authors corrected the drafts and revised the final version of the manuscript.

### Conflict of Interest Statement

The authors declare that the research was conducted in the absence of any commercial or financial relationships that could be construed as a potential conflict of interest.
